# Bone marrow derived-mesenchymal stem cell improves diabetes-associated fatty liver via mitochondria transformation in mice

**DOI:** 10.1186/s13287-021-02663-5

**Published:** 2021-12-11

**Authors:** Youkun Bi, Xuejun Guo, Mengqi Zhang, Keqi Zhu, Chentao Shi, Baoqi Fan, Yanyun Wu, Zhiguang Yang, Guangju Ji

**Affiliations:** 1 Institute of Biophysics, Chinese Academy of Sciences, Beijing, 100101 China; 2 Puyang Oilfield General Hospital, Affiliated to Xinxiang Medical College, Puyang city, 457000 Henan Province China; 3 University of Chinese Academy of Sciences, Beijing, 100049 China

**Keywords:** BMSCs, Diabetes, NAFLD, Mitochondrial, Mitochondrial transfer, Metabolism

## Abstract

**Background:**

Non-alcoholic fatty liver disease (NAFLD) has become a global epidemic disease. Its incidence is associated with type 2 diabetes mellitus (T2DM). Presently, there is no approved pharmacological agents specially developed for NAFLD. One promising disease-modifying strategy is the transplantation of stem cells to promote metabolic regulation and repair of injury.

**Method:**

In this study, a T2DM model was established through 28-week high-fat diet (HFD) feeding resulting in T2DM-associated NAFLD, followed by the injection of bone marrow mesenchymal stem cells (BMSCs). The morphology, function, and transfer of hepatocyte mitochondria were evaluated in both vivo and in vitro.

**Results:**

BMSC implantation resulted in the considerable recovery of increasing weight, HFD-induced steatosis, liver function, and disordered glucose and lipid metabolism. The treatment with BMSC transplantation was accompanied by reduced fat accumulation. Moreover, mitochondrial transfer was observed in both vivo and vitro studies. And the mitochondria-recipient steatotic cells exhibited significantly enhanced OXPHOS activity, ATP production, and mitochondrial membrane potential, and reduced reactive oxygen species levels, which were not achieved by the blocking of mitochondrial transfer.

**Conclusion:**

Mitochondrial transfer from BMSCs is a feasible process to combat NAFLD via rescuing dysfunction mitochondria, and has a promising therapeutic effect on metabolism-related diseases.

**Supplementary Information:**

The online version contains supplementary material available at 10.1186/s13287-021-02663-5.

## Introduction

The global prevalence of non-alcoholic fatty liver disease (NAFLD) has significantly increased in parallel with obesity [1]. NAFLD is characterized by diffuse fatty infiltration, accompanied by non-alcoholic steatohepatitis (NASH), and hepatic fibrosis [2]. The metabolic disorder of triglycerides and cholesterol results in the accumulation of massive lipid droplets in hepatocytes and subsequent hepatic steatosis, facilitating the follow-up of insulin resistance [3, 4]. The mechanistic basis of NAFLD and NASH is still not completely understood, and apart from insulin resistance, lipids, mitochondrial dysfunction, innate immunity, intestinal flora, genetic determinants, nutritional factors, and dietary structure also regulates the pathogenesis of NAFLD [5]. Many studies have reported a strong association between NAFLD and type 2 diabetes mellitus (T2DM), as over 70% of T2DM patients have NAFLD [6, 7]. The persistent imbalance of hepatic lipid uptake, de novo lipogenesis, and lipid clearance contributes to hepatic steatosis [8, 9]. Concomitant apoptosis and inflammation trigger the activation of stellate cells, leading to hepatic fibrosis, cirrhosis, and even hepatic carcinoma (HCC) [10].

Mitochondria play important roles in multiple metabolic processes, in addition to ATP generation, which is the most prominent [11]. The integrity of mitochondrial morphology and function is responsible for mediating apoptosis, ROS signaling, calcium signaling, and steroid synthesis [12, 13]. Both human and animal studies have described mitochondrial dysfunction in NAFLD and T2DM individuals in mitochondrial enzyme activity, abnormal mitochondrial morphology and number, and calcium activity [10, 14]. Mitochondrial quality control (MQC) is a complicated regulatory process that is crucial for maintaining cellular homeostasis [15]. MQC disorders are involved in the occurrence and development of NAFLD. Mitochondria dynamics are defined by fusion and fission, and are crucial for mitochondrial homeostasis, mitochondrial DNA (mtDNA) inheritance, and mitochondrial distribution. Sustained pathological stress, such as oxidative insult and lipotoxicity, overwhelms the MQC, greatly contributes to mitochondrial injury, which is detrimental to hepatocyte fate. Furthermore, MQC medication modulation has therapeutic benefits when combatting NAFLD, as described in many reports [16].

A promising strategy for the treatment of liver diseases is based on the application of stem cells or their secretions [17–19]. Among all stem cells, mesenchymal stem cells (MSCs) are characterized by their self-renewal potential and differentiation ability, leading to a good application in disease treatment [20, 21]. The therapeutic effect of MSCs mainly relies on paracrine signaling, differentiation, and inflammatory actions [22]. Studies have documented that MSCs can renew defective mitochondria via mitochondrial transfer between MSCs and diseased cells [23–25]. Transferred mitochondria cater for significant health benefits, as they correct the status of pathological cells by regulating MQC and maintaining macromolecular biosynthesis and cytoplasmic pools of NAD^+^ [25, 26]. Many studies have confirmed that mitochondrial transfer between MSCs and damaged tissue is a core event in tissue repair [27]. The positive effect of mitochondrial transfer was first clarified by rescuing aerobic respiration, for example, by increasing the oxygen consumption, membrane potential and intracellular ATP level [28]. MSC mitochondria transfer has been widely reported in the cardiovascular, respiratory, neurological, and renal systems [26, 29]. While not only is the therapeutic effect of BMSCs in NAFLD rarely reported, but mitochondria transfer between BMSCs and steatosis hepatocytes has not been clearly described. In the present study, we have firstly revealed that BMSCs can alleviate the steatosis in diabetes-associated NAFLD mice via mitochondria transformation.

## Materials and methods

### Experimental animals

All mouse-relevant procedures were performed according to the Guide for the Care and Use of Laboratory Animals published by the U.S. National Institutes of Health (NIH Publication No. 85-23, revised 1996)[30], and with the approval of the Institute of Biophysics Committee for Animal Care (Approval No. SYXK2019062). During acclimatization, 6-week-old male C57BL/6 strain mice purchased from Beijing Vital River Laboratory Animal Technology Co. Ltd were kept under standard housing conditions (12 h light/dark cycle, humidity 40–60%, temperature 22 °C) with ad libitum access to food and water. After adaptive feeding for one week, the animals were randomly divided into two groups fed with a growth and maintenance diet with 4% fat content (ND group) and a high-fat diet with 60% fat content (HFD group).

### Glucose and insulin tolerance test

After fasting for 16 h, tail tip blood of mice was subjected to blood glucose assay via Roche Glucose Meter and matching blood glucose test strip, followed by disinfecting the wound. Glucose tolerance test (GTT) was implemented to monitor the blood dynamic at 15, 30, 60, and 120 min, adopting the above blood glucose measurement, after glucose solution injection (Sigma, 1.5 g/kg, i.p.), before which the animals were fasted for 16 h; however, drinking was allowed. In the insulin tolerance test (ITT), tail venous blood from fasted mice for 4 h was monitored at 15, 30, 45, and 60 min, after insulin administration (Novolin R, 1 U/kg, i.p.). Before GTT and ITT, the glucose concentration of tail venous blood was measured as the initial reading.

### BMSC administration

The isolation, culture, and identification of BMSCs were completed as described in the Supplementary material. The certified BMSCs from 5 to passage 10 were selected as seed cells for intravenous injection.

BMSCs were digested with 0.25% trypsin, washed thrice with phosphate-buffered saline (PBS), and resuspended in saline. The cells were counted, and diluted to 1 × 10^6^ cells/mL in saline. For BMSC administration, the designed number of cells (1 × 10^7^ cells /kg body weight) suspended in 200 μL saline was gently injected via the caudal vein at 1 mL/min. Selfsame administration was performed in the animals of the control group, except for an equal volume of saline.

### Histological analysis

Animals were sacrificed at a specific time after the administration of BMSCs or an equal volume of saline.

After opening the chest, the liver was removed immediately, perfused with PBS to wash out the blood, and immersed in 4% paraformaldehyde for 48 h. The selected tissues were embedded in paraffin after dehydration in 75% ethanol for 12 h and then cut into 5 μm sections. The sections were deparaffinized and stained with hematoxylin and eosin (H&E) according to standard protocols. The Leica DFC300FX pathological image analysis system was used for image scanning and capture.

### Electron microscope imaging of isolated primary mHCs

The mHCs were isolated using the method described in the Supplementary Material. Thereafter, these cells were planted in a coverslip (Thermo, Massachusetts) for attachment. Well-attached cells were fixed in 2.5% glutaraldehyde at 4 °C overnight. The fixed mHCs were cut into ultrathin sections after performing a series of processing-based standard protocol [31]. A TEM (Thermo Fisher's Tecnai Spirit 120 kV) photographed the microscopic structure of the sections plated on the copper mesh.

### Establishment of steatotic cell model

Free fatty acids (FFAs) are a good choice for establishing a steatotic cell model. Well-growing HepG2 cells at 80% confluence were treated with FFA medium (DMEM medium with 10% FBS and 300 nM oleic acid as well as 300 nM sodium palmitate) for 36 h at 37 °C in an incubator. Oil Red O staining and TEM micrograph of cell thin sections supported the identification of the above established NAFLD model in vitro.

### Construction of mito-GFP cell line

The lentivirus infection method was used to establish cell lines stably expressing GFP in the mitochondria. Three plasmids, pLV-mitoGFP (Addgene, 44385), psPAX2 (Addgene, 12260), and pMD2.G (Addgene, 12259) were co-transfected into HEK293T cells at 80% confluency for 48 h, and the supernatant was collected. The lentivirus particles were prepared by filtering the supernatant with a 0.45 μm filter. The medium of well-cultured cells was replaced with serum-free DMEM (Gibco, New York) contained 10 multiplicity of infection (MOI) lentivirus and 10 μg/mL polybrene, and then cultured in an incubator at 37 °C for 12 h. The medium was replaced with a complete medium for 48 h of culture. Thereafter, these cells were digested with 0.25% trypsin to challenge the flow cytometry sorting to deliver signal cells with GFP expression to each well of a 96-well plate. A screening medium with 2.5 μg/mL puromycin was added to the above culture dish for cell expansion. After successive passages in the screening medium, cell line stably expressing GFP in the mitochondria was constructed. This study established HepG2 cells and BMSCs stably expressing GFP in mitochondria, named HepG2-mito-GFP, and BMSCs-mito-GFP, respectively.

### Detection of cellular ATP levels and oxygen consumption measurements

When the cells reached 90% confluency, they were ready to be lysed ATP production analysis using an enhanced ATP Assay Kit (Beyotime, China). The RLU or CPM levels were detected using a luminometer to evaluate ATP levels. The protein concentration of lysed samples was measured using Bradford assay kit (Sigma) to calculate the average ATP yield per μg of cell protein.

The oxygen consumption rate (OCR) was determined according to the protocols described previously using a Seahorse XF-96 extracellular flux analyzer (Seahorse Bioscience, Billerica). Approximately, 4 × 10^4^ mHCs were plated in 24-well plates and treated with F12/DMEM containing 1 mM bovine serum albumen (BSA)-conjugated oleate acid/palmitate acid (OA/PA) (Sigma) and incubated for 24 h before detection. Three independent repetitions were performed. The protein concentration in each well was quantified using the BSA assay according to the manufacturer’s instructions (Thermo). The OCR was normalized to the total protein concentration in each well.

### Mitochondrial transfer detection in vivo and vitro

The diabetic mouse model was anesthetized with a combination of ketamine and xylazine. A U-shaped incision was made, and the chest was opened to expose the liver. BMSCs-mito-GFP were mildly injected into T2D model mice (1 × 10^7^ cells/kg body weight in 200 μL saline) through caudal vein. One week later, the livers of BMSC-transplanted mice were harvested and subjected to imaging via IVIS® lumina III In Vivo Imaging System (PerkinElmer, USA). Then the removed livers were fixed with 4% paraformaldehyde, embedded in paraffin, and sectioned at 10 μm thickness. The sections were incubated with DAPI and photographed using a confocal microscope.

Thereafter, we isolated mHCs from T2DM mice and labeled them with 100 MitoTracker Red (Beyotime, C1049) at 37 °C for 30 min or not, and they were co-cultured with BMSCs-mito-GFP for 12 h. The live cell workstation was responsible for monitoring the mitochondrial movement. Also, BMSCs were isolated form 4–6-week-old male C57BL/6 strain mice and labeled with MitoTracker Red, and co-cultured with steatotic HepG2-mito-GFP for 12 h, followed by 2.5 μg/mL puromycin screen to kill BMSCs. The washed cells were fixed for confocal imaging.

Additionally, labeled mHCs isolated from HFD mice were co-cultured with BMSCs-mito-GFP with different cell proportions (11:1, 1:3, 1:5, 1:9, and 1:11) for 24 or 48 h, after which these cells were screened using 2.5 μg/mL puromycin. These cells were washed and subjected to 0.25% trypsin digestion. Flow cytometry was used to screen double-stained cells.

To investigate the treatment effect of mitochondrial transfer on steatotic cells, we pasted a 0.22 μm sterile membrane on the upper chamber of a transwell dish to block mitochondrial transfer, as described in Fig. 5D, and named this condition a mito-block. HepG2 cells were seeded at the bottom of the transwell dish with an FFA medium for 36 h. Thereafter, the FFA medium was replaced with complete medium, after which BMSCs were inoculated in the upper chamber for 24 h or 48 h to perform other tests. HepG2 cells in the lower layer were washed out with PBS buffer three times, and cell RNA was extracted to prepare cDNA using a reverse transcription kit (Vazyme, R211-01). mtDNA was detected using PCR analysis, and the human-specific gene ACTB was used as the control. The mtDNA was detected using the primers listed in Additional file 1: Table S1.

### Statistical analysis

The results were analyzed using a two-way ANOVA (multiple comparisons) or unpaired Student’s *t test*. Data are expressed as mean $\pm$ SE.* P* values considered statically significant were *P < 0.05, **P < 0.01, and ***P < 0.001.

## Results

### The treatment of bone marrow mesenchymal stem cell (BMSC) administration on T2D-associated NAFLD mice

**Fig. 1:**
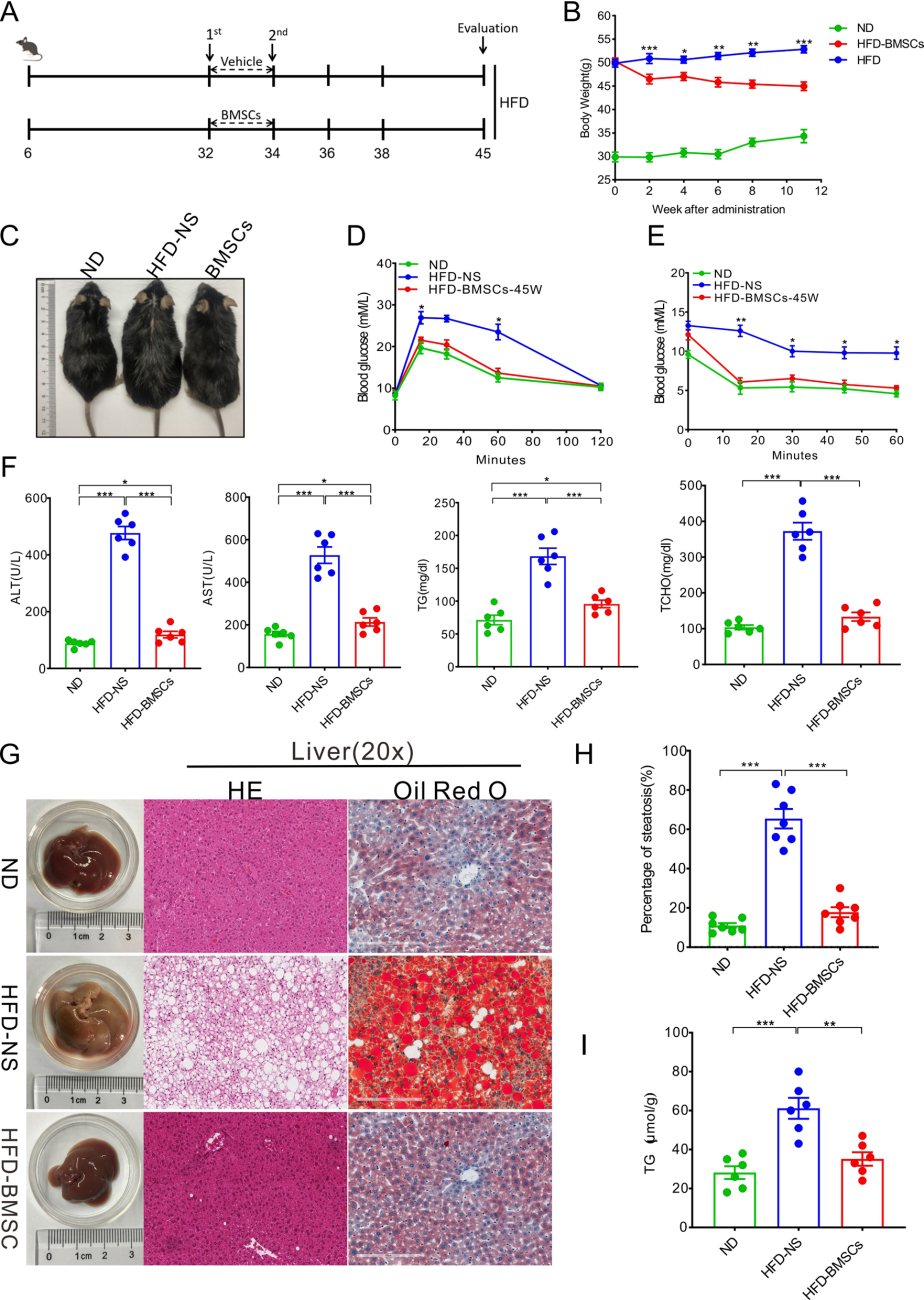
BMSC transplantation rescues T2D-associated NAFLD. **A** Experimental design for establishing T2D model and assessing the BMSC treatment on T2D-associated NAFLD. Six-week C57BL/6 J mice were fed with HFD throughout the trial. Two BMSC injections were performed at 32nd and 34th week. Additionally, two vehicle injections (normal saline) were set as control. **B** The dynamic monitoring of mice after BMSC or vehicle administration (started at 34th week). *N* = 10 animals per group. **C** Representative images of ND mice and T2D-associated NAFLD mice administered with vehicle or BMSCs. The images were photographed at 45th week. **D** GTT and **E** ITT analyses were carried out to evaluate the sensitivity of mice at 45th week to glucose and insulin. *N* = 6 animals per group. **F** Serum biochemical assay of mice at 45th week. Sera of mice at 45th week were isolated to detect ALT, AST, TG, and TCHO levels. *N* = 6 animals per group. **G** Representative images of gross liver and assays for HE and oil red O-stained liver sections. **H** Image J software was utilized to assay steatosis degree of liver. *N* = 7 animals per group. **I** Homogenized liver tissues were subjected to TG detection. *N* = 6 animals per group. All statistical data are represented as means ± s. *P < 0.05; **P < 0.01; ***P < 0.001. All imaging was performed and analyzed in a blinded fashion

T2D mice were selected from mice fed with HFD from 6th to 32nd week based on the diagnostic criteria for type 2 diabetic mice promulgated by the World Health Organization (WHO) [32] (Fig. 1A and Additional file 1: Fig. S1). Thereafter, they were randomized to either the vehicle or the treatment of identified and purified BMSCs (Additional file 1: Fig. S2) at 32nd week and 34th week. The monitoring of dynamic weight after treatment revealed that BMSC injection notably reduced the weight of HFD mice (Fig. 1B). The morphology photograph at 45th week highlighted the weight correction of BMSCs (Fig. 1C). However, BMSCs did not reverse the weight of HFD mice to normal levels. GTT and ITT analyses were performed at the 45th week, and the results together showed that HFD mice regained sensitivity to glucose and insulin (Fig. 1D, E). Thereafter, the sera from the 45-week-old mice were subjected to biochemical testing. BMSC treatment reversed the abnormally elevated ALT, AST, TG, and TCHO levels in HFD mice; however, these levels were still higher than the normal levels (Fig. 1F).

T2D is known to be closely related to obesity and NAFLD [6]. To investigate the effects of BMSC treatment on NAFLD, we performed a histopathological analysis of the liver at the 45th week (11 weeks after BMSC administration). We observed severe steatosis and significantly increased TG levels in the liver of HFD mice (Fig. 1G–I). BMSC administration completely reversed the steatosis in the liver of HFD mice, and this phenomenon was observed in all examined mice (total *n* = 10). Additionally, brown adipose tissue (BAT) is physiologically crucial for energy metabolism. Pathological analysis revealed that HFD was an important driving factor for converting BAT into white adipose tissue (WAT). BMSCs also alleviated the above condition in the BAT of T2D mice; however, BAT did not return to normal levels in mice of the ND group (Additional file 1: Fig. S3).

### BMSCs improve the dysfunction of mitochondria in mHCs

**Fig. 2:**
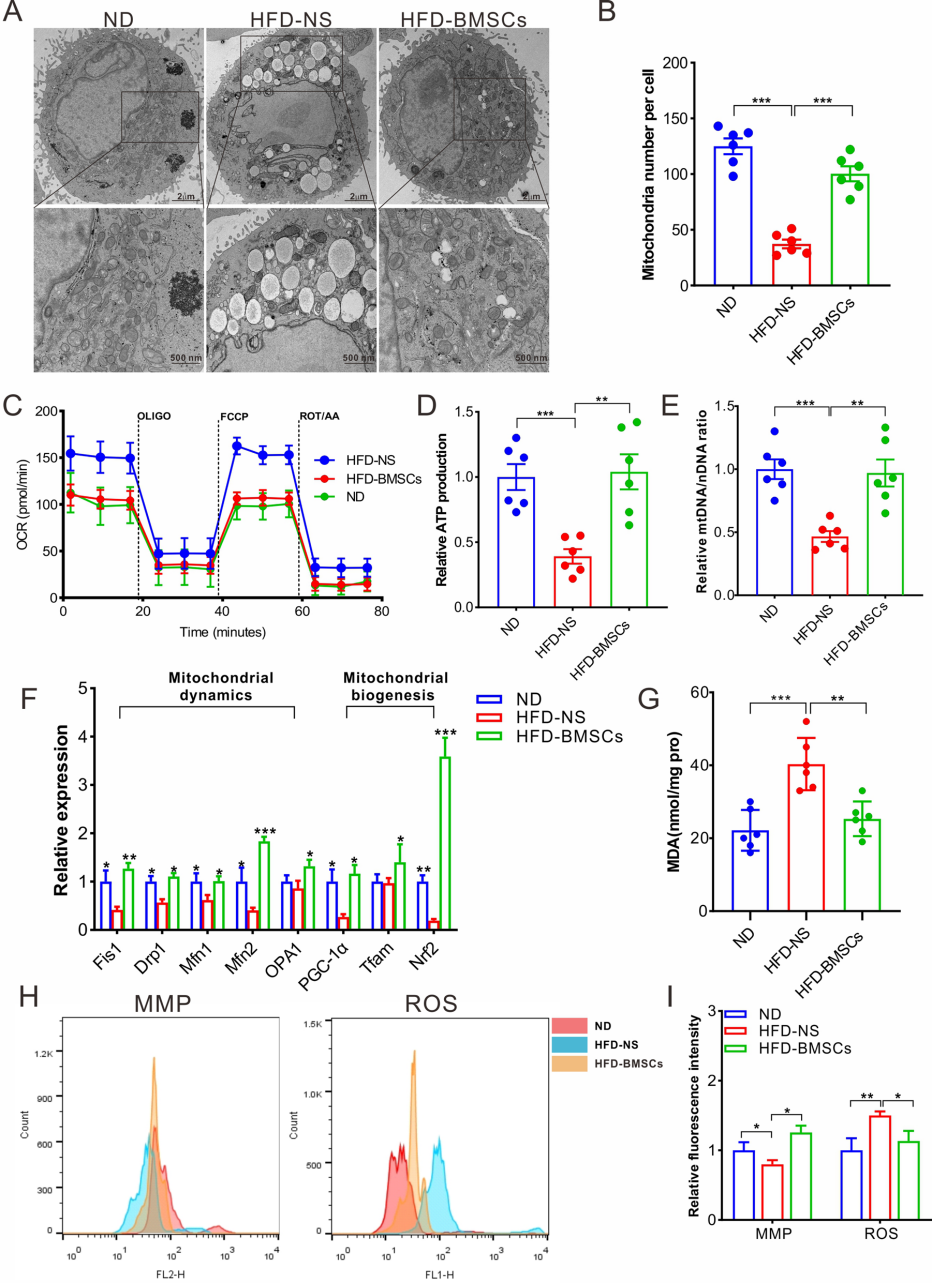
BMSC transplantation rescues mitochondrial dysfunction. **A** TEM micrographs of isolated mHCs from ND mice and T2D-associated NAFLD mice administered with vehicle or BMSCs at 45th week. **B** The average number of mitochondria in each cell. The statistics are from random 25 cell fields per mice and 6 mice per group. **C** OCR analysis of isolated mHCs from ND mice and T2D-associated NAFLD mice administered with vehicle or BMSCs at 45th week. The mHCs come from 6 mice per group. **D** The ATP detection of isolated mHCs. The mHCs were isolated from 6 mice per group. **E** Real-time PCR describes the mtDNA/nDNA ration according to the previous report [60]. Relative quantification was performed on 6 animals per group. **F** The mRNA expression level of mitochondria-related genes in terms of dynamics and biogenesis. The RNA was extracted from homogenized liver tissues of 6 mice per group. **G** MDA detection of homogenized liver tissues of 6 mice per group. **H**, **I** MMP and ROS detection of mHCs from 6 mice per group. All statistical data are represented as means ± s. *P < 0.05; **P < 0.01; ***P < 0.001

Mitochondria play a crucial role in physical metabolism [25, 26]. It is also well known that a long-term HFD is responsible for mitochondrial dysfunction [33]. To investigate the effect of BMSCs on mitochondria in NAFLD mHCs, ultrathin sections of isolated mHCs were subjected to electron microscopy imaging for mitochondrial morphology observation. Micrographs revealed that HFD promoted the accumulation of lipid droplets in mHCs. The mitochondrial number significantly decreased, accompanied by morphological abnormalities such as concentration, the disappearance of cristae, and swelling (Fig. 2A, B). OCR value assessment of isolated mHCs clarified the elevation in oxygen consumption in HFD mice (Fig. 2C) in terms of base, maximal, and spare respiratory levels (Additional file 1: Fig. S4). The decrease in unit ATP production (Fig. 2D) and mtDNA copy number (Fig. 2E) further supported mitochondrial dysfunction in mHCs of HFD mice. BMSC treatment restored mitochondrial abnormalities to normal levels in multiple terms, such as morphology, mtDNA copy number, OCR, and unit ATP production (Fig. 2).

MQC is recognized by changing the structure and shape of mitochondria through fusion and fission processes in response to changes in the energy demand and supply [15]. Pathological stress disrupts mitochondrial biogenesis and dynamics, accompanied by corresponding changes in the expression of crucial genes, especially Fis1, Drp1, Mfn1, Mfn2, OPA1, PGC-1α, Tfam, and Nrf2 [34]. Liver tissues were homogenized for RNA extraction and cDNA preparation. Real-time polymerase chain reaction (PCR) was used to assess the changes in the expression of MQC-relevant genes using the primers listed in Additional file 1: Table S2. The results indicated that expression of nearly all test genes significantly decreased in the T2D model, and BMSC treatment corrected their expression to normal levels, and even to the levels lower than the normal levels (Fig. 2F).

Additionally, other indicators were used to evaluate mitochondrial activity. The malondialdehyde (MDA) level is an important parameter that reflects antioxidant potential. High MDA levels attenuate the activities of the respiratory chain complex and key enzymes in mitochondria, and continuous elevation of MDA level destroys the integrity of the mitochondrial membrane. Metabolic disorders result in a sharp rise in ROS levels. Excess ROS level increases the permeability of the mitochondrial double-layer membrane, which is responsible for follow-up adverse events, such as mitochondrial membrane potential (MMP) decrease, mitochondrial dysfunction, and apoptosis [35, 36]. This study found a significant increase in MDA and ROS levels in HFD mice compared to those in ND mice. MMP decline was also confirmed in the T2D model owing to the long-term HFD treatment. Conversely, BMSC treatment restored abnormal MDA, ROS, and MMP levels back to normal levels (Fig. 2G–I).

### BMSC treatment directly enhance the mitochondrial activity in vitro

**Fig. 3:**
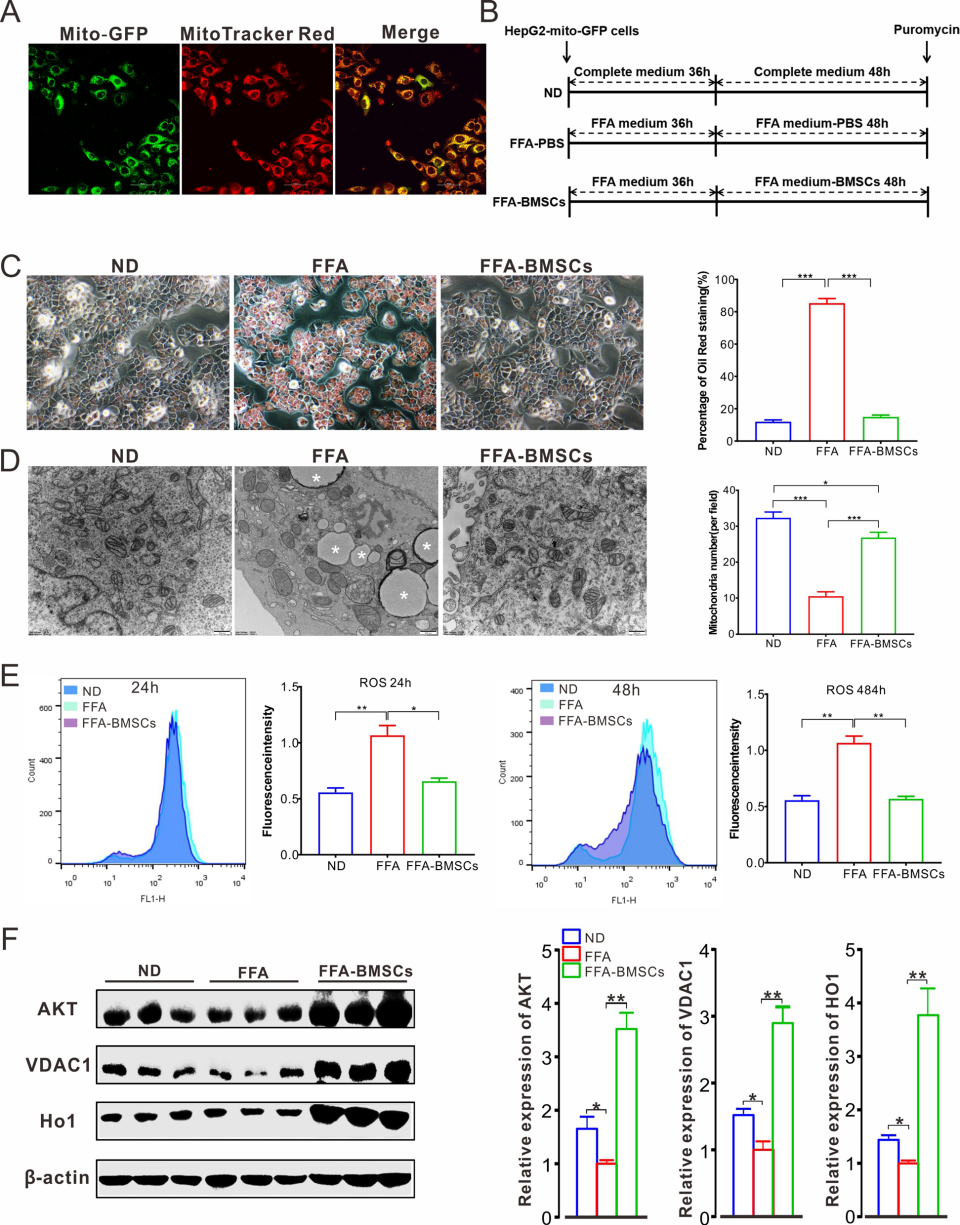
BMSC intervention directly enhances the mitochondrial activity in vitro. **A** The establishment of HepG2-mito-GFP cell lines. The mitochondria were stained with MitoTracker Red. Scare bar represents 25 μm. **B** Trial design for establishing steatotic cell model and assessing the BMSC intervention effect: well-growing HepG2-mito-GFP cells were fed with FFA medium for 36 h, and then BMSCs were added for 48 h co-cultivation, followed by puromycin screening. Cells fed with complete medium were set as control. **C** Oil Red O stain detection of treated cells in **B** and the quantification of Oil Red O staining area. Three independent replicates were performed. **D** Representative TEM imaging of treated cells in **B** and the quantification of average mitochondrial number in the field at 500 nm. Big droplets are labeled with asterisk. Twenty-five fields per group were taken into consideration. **E** Total ROS level was detected via FCM and quantitative analysis at 24 h and 48 h. Three independent replicates were performed. **F** WB assay of target protein level. The relative expression level was determined by densitometry and normalized to β-actin levels. All statistical data are represented as means ± s. *P < 0.05; **P < 0.01; ***P < 0.001

The 10th passage cells in good condition were planted in a confocal dish and incubated with 100 nM Mito-Tracker Red (Beyotime) for 20 min at 37 °C. Confocal imaging revealed that green fluorescence in HepG2-mito-GFP had a perfect overlap with MitoTracker Red (Fig. 3A), which indicated the stable expression of GFP in mitochondria. HepG2-mito-GFP cells were plated at an appropriate density in a 6-well plate and cultured with free fatty acid (FFA) medium for 36 h at 37 °C to induce steatosis (Fig. 3B). Oil Red O staining and transmission electron microscope (TEM) micrograph of thin cell sections together indicated that there was a large accumulation of lipid droplets in steatotic cells (Fig. 3C). TEM micrographs also showed that FFA additives contributed to mitochondrial abnormalities, such as swelling and a sharp decline in the mitochondrial number (Fig. 3D).

To investigate the effect of BMSCs on steatotic cells, HepG2-mito-GFP cells were cultured in FFA medium for 36 h and then co-cultured with BMSCs for another 24 h or 48 h, followed by puromycin screening to kill viable BMSCs. The surviving cells were subjected to Oil Red O staining and TEM imaging. Both intuitively displayed BMSC treatment-corrected steatosis in terms of a significantly decreased number of lipid droplets and mitochondrial recovery (Fig. 3B, C). Additionally, these cells were subjected to ROS detection using flow cytometry. The results revealed that BMSCs positively regulated the inhibition of total cell ROS production induced by lipotoxicity at 24 h and 48 h (Fig. 3E). Similarly, the elevated mitochondrial ROS level in steatotic cells was also significantly attenuated after BMSC intervention at 48 h; however, it did not show a remarkable reduction at 24 h (Additional file 1: Fig. S5).

Previous studies have reported that some genes play a crucial role in regulating mitochondrial function. Among them, AKT participates in the determination of cell fate by restraining apoptosis induced by preventing cytochrome C release from the mitochondria [37]. VDAC1, a voltage-dependent anion channel, is closely related to the communication of mitochondria and cytoplasm [38]. HO-1 signaling protects cells from oxidative stress and inflammatory responses. Therefore, we performed western blot analysis targeting cell lysates in each group [39]. The results revealed that FFA feed significantly contributed to lowering the gene expression of AKT, VDAC1, and HO1 compared to that of ND. BMSC treatment further elevated the expression relative to the ND level (Fig. 3F).

### BMSCs rescue the abnormal calcium activity of steatotic cells

**Fig. 4:**
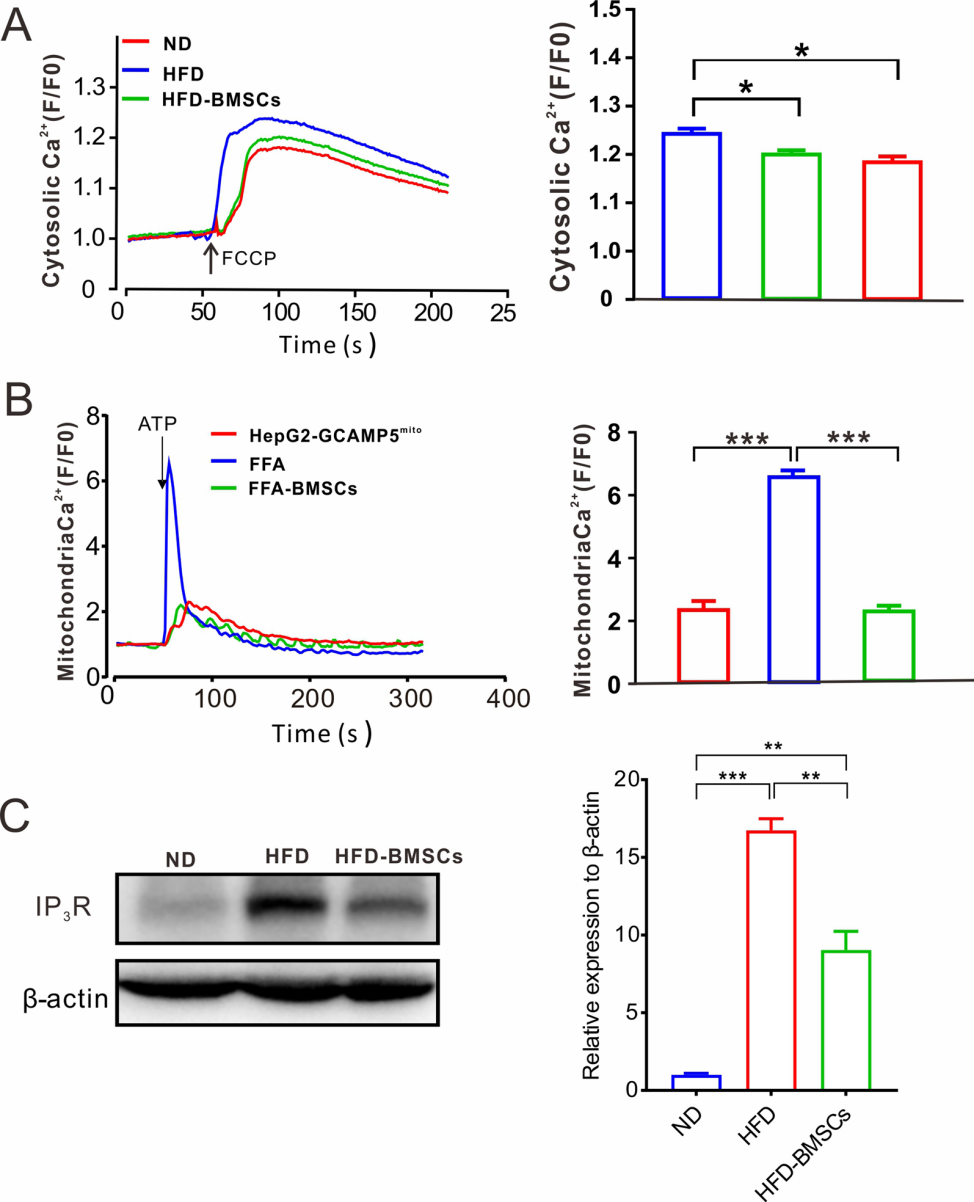
BMSCs rescue the abnormal calcium activity of steatotic cells. **A** Representative Ca^2+^ transients induced by FCCP form linescan images recorded from isolated mHCs of ND (red), HFD (blue), and HFD-BMSCs (green) mice. **B** Representative Ca^2+^ transients induced by ATP form linescan images recorded from HepG2-GCAMP^mito^ cells treated with complete medium (red), FFA medium (blue), and FFA medium combined with BMSCs (green). **C** The detection of IP3R expression in the liver of ND, HFD, and HFD-BMSCs mice. The relative expression was determined by densitometry and normalized to β-actin levels. All trials above were performed with three independent replicates, and the statistical data are represented as means ± s. *P < 0.05; **P < 0.01; ***P < 0.001

The liver is a major metabolic organ that controls gluconeogenesis, glycogen storage, lipogenesis, and cholesterol and bile acid metabolism [1, 2]. Alteration of Ca^2+^ at the cellular and organelle levels of the liver directly affects hepatic glucose production and lipogenesis [40, 41]. To investigate the effect of BMSCs on the calcium activity of steatotic cells, we measured the relative calcium concentration in the cytoplasm and mitochondria. First, we isolated primary hepatocytes from HFD mice, which were loaded with calcium probe fluo-2 for 10 min at room temperature, and confocal fluorescence intensity dynamic curves were recorded. The results indicated that the cytoplasmic calcium activity of steatotic cells showed higher sensitivity to carbonyl cyanide *p*-trifluoromethoxyphenylhydrazone (FCCP) stimulation, an oxidative phosphorylation uncoupler, than that of normal mHCs. BMSC treatment reduced the calcium load in the cytoplasm of steatotic cells to normal levels (Fig. 4A). Thereafter, we established that HepG2 cells stably expressed GCAMP5 in mitochondria, named HepG2-GCAMP5mito. These cells were treated with FFA medium for 36 h for the next mitochondrial calcium detection. The calcium signal curve showed a steep rise after the steatotic cells were stimulated with ATP. This phenomenon was corrected to normal levels after co-culture with BMSCs (Fig. 4B).

IP3R directly regulates calcium signal transduction and mediates triglyceride storage in hepatocytes [42]. The activation of the IP3R signaling pathway is responsible for the elevated calcium concentration and steatosis in hepatocytes [43]. This study detected the expression of IP3R1 in the liver of each group of mice. The results indicated that BMSCs significantly reduced IP3R1 expression in HFD mice (Fig. 4C).

### Mitochondrial transfer from BMSCs to steatosis cells in vitro and in vivo

**Fig. 5:**
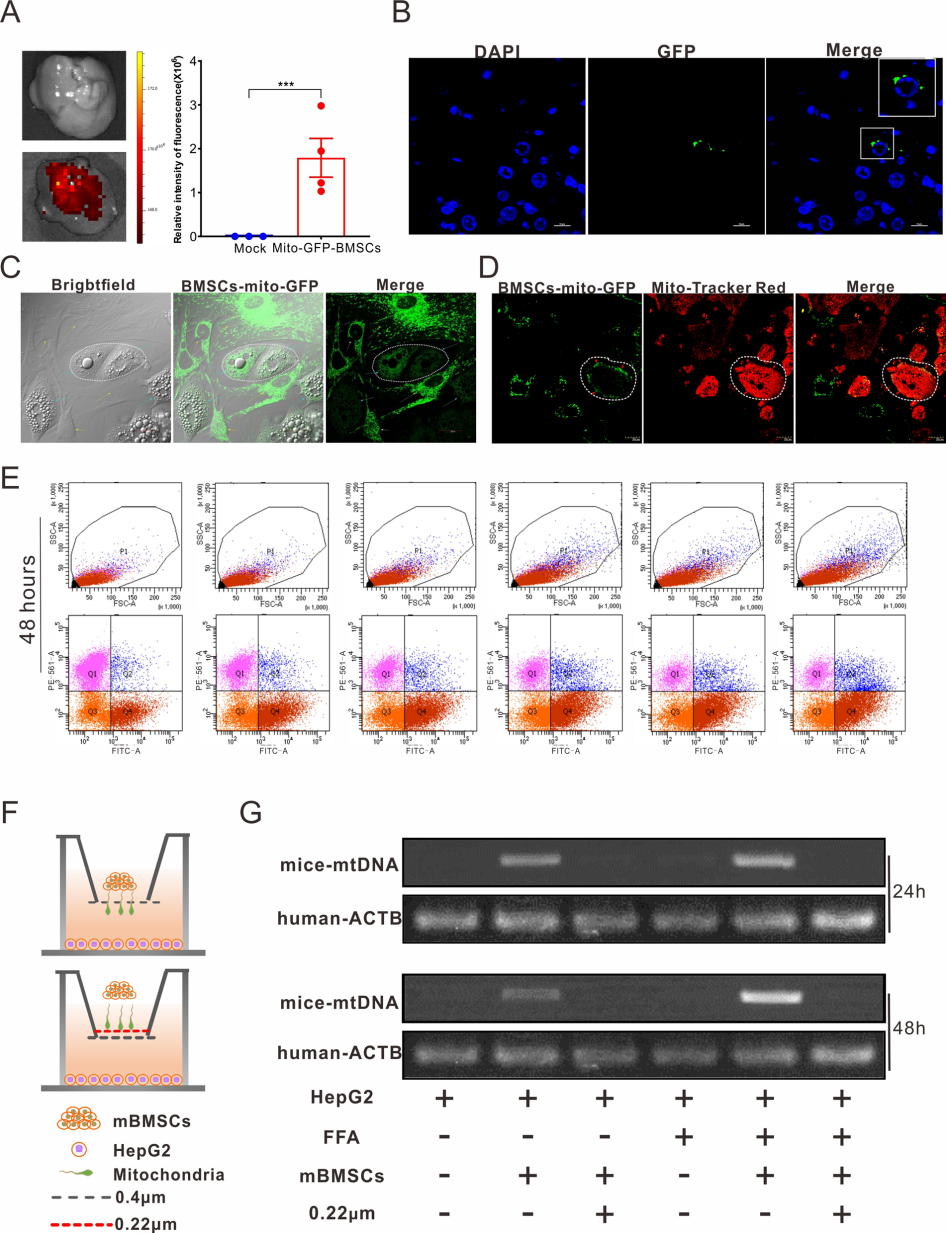
Mitochondria transfer from BMSCs to steatotic cells. **A** The detection of GFP signaling in liver after BMSCs-mito-GFP for one week using an in vivo imaging system. All statistical data are represented as means ± s.***P < 0.001. **B** The detection of mito-GFP signaling from BMSCs in liver. Blue, nucleus; Green, mito-GFP. Scale bar = 10 μm. **C** Representative micrographs of isolated mHCs from T2DM mice and BMSCs-mito-GFP after co-cultivation for 12 h. Green, BMSCs-mito-GFP; Red, MitoTracker Red. Scale bar = 20 μm. **D** Representative micrographs of labeled BMSCs and HepG2-mito-GFP after co-cultivation for 12 h. Green, HepG2-mito-GFP; Red, MitoTracker Red. Scale bar = 20 μm. **E** Flow cytometry detects the double-stained cells. Labeled mHCs isolated form HFD mice were co-cultured with BMSCs-mito-GFP with different cell proportions (11:1/1:3/1:5/1:9/1:11) for 48 h, after which these cells were screened using 2.5 μg/mL puromycin. Q2 represents the double-stained cells. **F** Schematic diagram of experimental design to verify mitochondrial transfer. HepG2 cells were planted at the bottom of a transwell dish with FFA medium for 36 h. Then, the FFA medium was replaced with complete medium, after which BMSCs were inoculated at the upper chamber with 0.4 μm or 0.22 μm filter membrane for 24 h or 48 h. **G** The mtDNA detection via gel electrophoresis at 24 h and 48 h

These results indicated that BMSC treatment suppressed steatosis by correcting mitochondrial disorders. This study hypothesized that the mitochondria of BMSCs directly transfer into adjacent cells to combat pathological stress. Live imaging observed GFP signaling in liver after BMSCs-mito-GFP injection for one week (Fig. 5A). Confocal microscope imaging of paraffin sections also revealed green fluorescence accumulation around the hepatocyte nucleus (Fig. 5B), which initially verified the transfer of mitochondria from BMSCs to hepatocytes. Next, the isolated mHCs from T2DM mice were labeled with MitoTracker-Red and then co-cultured with BMSCs-mito-GFP. Dynamic monitoring in 12 h intuitively described the mitochondrial transfer from BMSCs-mito-GFP to adjacent hepatocytes (Fig. 5C and Video S). The micrograph displays the overlap of green and red fluorescence, which indicated the HepG2-mito-GFP received the mitochondrial form labeled BMSCs (Fig. 5D).

Flow cytometry corroborated mitochondrial metastasis. MitoTracker Red-labeled mHCs isolated from HFD mice were co-cultured with BMSCs-mito-GFP with different cell proportions (11:1/1:3, 1:5, 1:9, and 1:11) for 24 or 48 h. Double-stained cells were differentiated using flow cytometry. In the same proportion of isolated mHCs, the number of double-stained cells positively correlated with the seed number of BMSCs. Increased co-culture time also contributed more to the increase in the number of double-stained cells (Fig. 5E and Additional file 1: Fig. S6). Moreover, we investigated mitochondrial transfer in vitro. HepG2 cells and BMSCs were seeded at the bottom and top chambers of a transwell device, respectively, with 0.4 μm or 0.22 μm membrane for 24 h or 48 h culture (Fig. 5F). RNA from HepG2 cells in the lower layer was extracted for cDNA preparation. mtDNA was detected using PCR analysis, and the human-specific gene ACTB was used as the control. The electrophoresis bands indicated that mtDNA from BMSCs appeared in HepG2 cells regardless of FFA feed. However, this situation was blocked by a 0.22 μm membrane pasted to the upper chamber (Fig. 5G). This comprehensively supports the mitochondrial transfer from BMSCs to HepG2 cells.

### Mitochondrial transfer dominates the rescue of steatotic cells not paracrine signaling

**Fig. 6:**
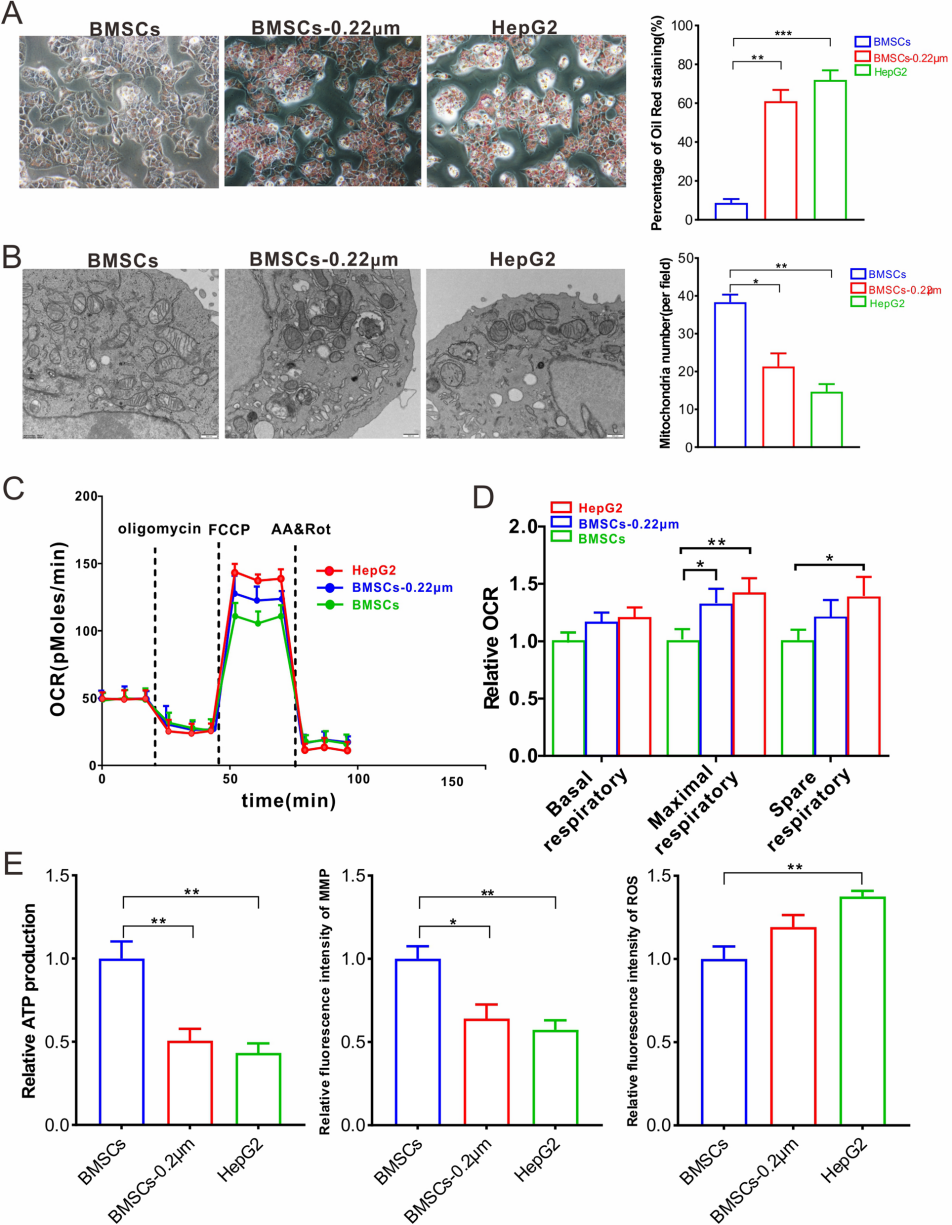
Mitochondrial transfer dominates the rescue of steatotic cells over paracrine signaling. Cells were processed as the description in Fig. 5F. **A** Representative micrographs of Oil Red O-stained cells and the quantification of stained area. **B** TEM imaging of differently treated cells, and the quantification of average mitochondrial number at 500 nm. Twenty-five fields per group were taken into consideration. **C** OCR assay of differently treated cells. **D** Relative OCR comparison in terms of basal, maximal, and spare respiratory levels. **E** Detection of relative ATP, MMP, and ROS levels in differently treated cells. ΒΜSCs-group and BMSCs-0.22 μm-group indicate BMSCs were planted at the upper chamber with 0.4 μm or 0.22 μm filter membrane, respectively. HepG2-group means HepG2 cells were planted at the upper chamber with 0.4 μm filter membrane. All trials above were performed with three independent replicates, and the statistical data are represented as means ± s. *P < 0.05; **P < 0.01; ***P < 0.001

Many studies have reported that BMSC paracrine signaling plays a crucial role in combating pathological stress [22]. To investigate the treatment effect of mitochondrial transfer to steatotic cells, we performed trials to verify the importance of mitochondrial transfer in alleviating steatosis. Oil Red O staining and cell ultrathin sections were used to detect steatosis and mitochondria. The results revealed that mito-block failed to rescue steatosis; however, there was still a slight improvement compared to the control (Fig. 6A). SEM micrographs showed that mito-block also could not correct the abnormal mitochondria, including morphology and number, in steatotic cells (Fig. 6B). Thereafter, the OCR analysis indicated that the mito-block was less effective than the unblocked condition. The monitoring of maximal and spare respiratory levels both showed a significant decrease in BMSC administration compared to those of the mito-block condition (Fig. 6C, D). Moreover, unit ATP production and MMP levels did not rebound when mitochondrial transfer from BMSCs was blocked. Under mito-block conditions, ROS levels did not decrease to normal levels (Fig. 6E). These results show that mitochondrial transfer from BMSCs dominates the rescue of steatotic cells, not paracrine signaling.

## Discussion

NAFLD has become a global epidemic disease associated with multiple metabolic disorders, such as obesity and T2DM [3, 4]. The coexistence of NAFLD and T2DM creates a worse metabolic profile, resulting in an increased risk of cardiovascular diseases, advanced fibrosis or cirrhosis, and even HCC [44, 45]. NAFLD and T2DM can be improved by dietary changes, increasing physical activity, and other treatment options such as metformin [46]. Moreover, there is currently no approved pharmacological agent specially developed for NAFLD. At this time, BMSCs have increased interest through the effect on chronic liver injury caused by decreasing apoptosis and immune imbalance [47, 48]. In a rodent model of fulminant hepatic failure and liver fibrosis, BMSC administration notably improved the pathological injury of the liver [49]. Additionally, BMSCs reversed HFD-induced NAFLD by suppressing CD4+ T lymphocytes in mice [50]. However, there are no reports on evaluating the treatment effect of BMSCs on NAFLD in T2DM. To date, only two studies have detailed the therapeutic effect of BMSCs on the HFD-induced rodent NAFLD model [50, 51]. However, neither has described the role of BMSCs in T2DM-associated NAFLD. This study established a T2DM model through 26-week HFD feeding, leading to severe liver steatosis and disordered glucose metabolism. Thereafter, the mice received two BMSC injections for the treatment evaluation. Particularly, mice were not withdrawn from HFD feeding after BMSC transplantation. Comprehensive analysis revealed that BMSC transplantation alleviated the collapsed liver function, hepatic steatosis, and lipid accumulation supported by reversing the evaluated AST and ALT to normal levels, lowering the AST/ALT ratio, and relieving histological lesions in the liver tissue. Moreover, BMSCs improved the disturbed glucose and lipid metabolism demonstrated by restoring GTT and ITT levels to normal levels and lowering the abnormal TG, TC, LDL, and HDL levels. It is worth mentioning that the GTT level had an exception after BMSC infusion for 4 weeks compared to the detection levels at 36th and 45th week. Although BMSC administration significantly reduced the body weight compared to that of diabetic obese mice, the mice were still significantly heavier than normal. The stubborn fat accumulation in the abdomen of the therapy group may be attributed to the above results. Therefore, it is necessary to further increase the therapeutic effect after increasing the total number of injections and the number of injected cells.

Mitochondria serve as cellular power plants that generate ATP by utilizing substrates derived from fat and glucose [11]. Hepatocytes have robust mitochondrial reserves. An increasing number of studies have confirmed that mitochondrial dysfunction is closely related to NAFLD pathogenesis, which raised the viewpoint that NAFLD is a mitochondrial disease [16, 52]. In this study, electronic microscopy showed that mitochondria in NAFLD were large and swollen and scarce in number, and the cristae disappeared. The alteration of mitochondrial morphology resulted in inefficient oxygen use, decreased ATP production, total mtDNA, mRNA levels of MQC-related genes, and MMP, and increased ROS levels in hepatocytes. All these effects were effectively reversed by BMSC transplantation. Based on the evidence reported previously, our study confirms that mitochondrial dysfunction is a primary mechanism for NAFLD development. Mitochondrial dysfunction results in fat accumulation and leads to ROS generation, contributing to NAFLD progression. Mitochondrial correction may be an optimal treatment strategy for NAFLD.

The mitochondrial transfer enables the replacement of damaged mitochondria in diseased cells to reduce ROS production, introduce new exogenous bases for mtDNA repair, upregulate ATP yield, and slow down calcium influx [23, 28, 53]. Ismlam et al. first reported that BMSCs could supply robust mitochondria to alveolar epithelial cells in a rodent model of E. coli lipopolysaccharide-induced acute lung injury [54]. In our study, we observed that the delivery of mitochondria from BMSCs into steatotic cells increased OXPHOS activity and ATP levels, which in turn maintained cellular bioenergetics and recovered hepatocyte function. By contrast, blocking mitochondrial transfer deterred the protective effects of BMSCs. Classical mechanisms of paracrine release of cytokines and growth factors are related to rescuing damaged cells. However, mitochondria-free paracrine effect contributed less to rescue steatotic cells in vitro within 48 h. Hence, we speculate that mitochondrial transfer from BMSCs, not paracrine signaling, dominates the rescue of damaged cells. Several studies have also stated that the horizontal transfer of mitochondria from one cell to another rescues the damaged cells with healthy ones, such as MSCs [54, 55]. Additionally, the natural intercellular mitochondrial transfer of MSCs enhances the immune response of macrophages. Rustom et al. first described the intercellular organelle transport between cells [56], from which multiple studies showed that MSCs are the best cells to transfer mitochondria [28, 54, 57]. Considering the reliable benefits of natural mitochondrial transfer, it is urgent to better understand its mechanism to facilitate the artificial replication of this process.

Our investigation is the first to report that BMSCs correct diabetes-associated NAFLD and emphasized that the nature of the cellular mitochondrial transfer is crucial for the above process to rescue steatotic cells. Nevertheless, there are still many issues that need to be resolved. Increasing reports support paracrine participants in immune regulation and tissue regeneration in NAFLD [58], and further investigation should focus on the crosstalk between BMSCs and other mitochondria-recipient cells such as macrophages and stellate cells. Additionally, the mitochondrial morphology and its plasticity allow it to be transported by subcellular transport mechanisms such as tunneling nanotubes and extracellular vesicles [57, 59]. A better understanding of the process of mitochondrial transfer from BMSCs is beneficial for promoting the efficiency of mitochondrial transfer. The techniques currently in use today include con-incubation of isolated mitochondria and recipient cells and physical approaches to induce integration. Another priority is to determine the optimum quality of mitochondria for tissue repair by improving the current artificial transfer methods. Mitochondria are more than cell power plants. Therefore, we should continue to develop their application in medicine.

## Conclusion

In summary, our study first revealed that BMSCs can alleviated the steatosis in diabetes-associated NAFLD mice. Moreover, mitochondrial transfer from BMSCs contributed greatly to combating steatosis via revising dysfunction mitochondrial, and has a promising therapeutic effect on NAFLD.

## Supplementary Information

**Additional file 1: Fig. S1**. Establishment of T2D-associated NAFLD mouse model. (A) The body weight monitoring of mice fed with HFD or ND from 6th week to 32nd week. (B) The detection of serum insulin at 32nd week. (C) The GTT and ITT assay at 32nd week. (D) The detection of serum ALT and AST at 32nd week. All statistical data are represented as means ± s. *P < 0.05; **P < 0.01; ***P < 0.001. **Fig. S2**. The identification of BMSCs. (A) The representative micrographs of the 4th passage BMSCs (a1); Adipogenic differentiation of the 6th passage BMSCs (Oli Red O stain of lipid droplets); Osteogenic differentiation of BMSCs (Alizarin Red stain for calcium). Scale bars 10 μm. (B) Representative flow cytometry analysis of cell-surface markers in the 6th passage BMSCs. All BMSCs expressed cell markers included CD73 and CD90.2, however negative for CD34 and CD45. **Fig. S3**. Representative images of HE stained BAT sections. ND, normal diet; HFD, high fatty diet; HFD-BMSC, high fatty diet mice with BMSC administration. **Fig. S4**. The calculations of mean OCR of the isolated mHCs at different stage corrected to basal OCR, maximal respiratory and spare respiratory capacity. The mHCs were isolated from ND, HFD-NS, and HFD-BMSCs mice at 45th week. All statistical data are represented as means ± s. *P < 0.05; **P < 0.01. **Fig. S5**. The flow cytometry analysis of mitochondrial ROS of different cells at 24 h and 48 h, respectively. HepG2-mito-GFP cells were cultured in FFA medium for 36 h and then co-cultured with BMSCs (FFA-BMSCs group) or not (FFA group) for another 24 h or 48 h. All statistical data are represented as means ± s. **P < 0.01. **Fig. S6**. Flow cytometry detects the double-stained cells. Labeled mHCs isolated form HFD mice were co-cultured with BMSCs-mito-GFP with different cell proportions (11:1/1:3/1:5/1:9/1:11) for 24 h, after which these cells were screened using 2.5 μg/mL puromycin. Q2 represents the double-stained cells. **Table S1**. Sequence information for the analysis of mtDNA/nDNA ratio. **Table S2**. Sequence information of MQC-relevant genes.

## Data Availability

All datasets used and/or analyzed during the current study are available from the corresponding author on reasonable request.

## Abbreviations

BMSC: Bone marrow mesenchymal stem cell
NAFLD: Non-alcoholic fatty liver disease
T2DM: Type 2 diabetes mellitus
HFD: High-fat diet
MMP: Mitochondrial membrane potential
ROS: Reactive oxygen species
HCC: Hepatic carcinoma
MQC: Mitochondrial quality control
mtDNA: Mitochondrial DNA
WHO: World Health Organization
mHCs: Mouse hepatocytes
OCR: Oxygen consumption rate
MDA: Malondialdehyde
FFA: Free fatty acid
TEM: Transmission electron microscope
FCCP: *p*-Trifluoromethoxyphenylhydrazone
