# Supplementary material for: Bone marrow derived-mesenchymal stem cell improves diabetes-associated fatty liver via mitochondria transformation in mice
Source: Stem Cell Res Ther. 2021 Dec 11;12:602. doi: 10.1186/s13287-021-02663-5 (PMC8665517; doi:10.1186/s13287-021-02663-5)
Supplement: Supplementary file 1 — Additional file 1: Fig. S1. Establishment of T2D-associated NAFLD mouse model. (A) The body weight monitoring of mice fed with HFD or ND from 6th week to 32nd week. (B) The detection of serum insulin at 32nd week. (C) The GTT and ITT assay at 32nd week. (D) The detection of serum ALT and AST at 32nd week. All statistical data are represented as means ± s. *P < 0.05; **P < 0.01; ***P < 0.001. Fig. S2. The identification of BMSCs. (A) The representative micrographs of the 4th passage BMSCs (a1); Adipogenic differentiation of the 6th passage BMSCs (Oli Red O stain of lipid droplets); Osteogenic differentiation of BMSCs (Alizarin Red stain for calcium). Scale bars 10 μm. (B) Representative flow cytometry analysis of cell-surface markers in the 6th passage BMSCs. All BMSCs expressed cell markers included CD73 and CD90.2, however negative for CD34 and CD45. Fig. S3. Representative images of HE stained BAT sections. ND, normal diet; HFD, high fatty diet; HFD-BMSC, high fatty diet mice with BMSC administration. Fig. S4. The calculations of mean OCR of the isolated mHCs at different stage corrected to basal OCR, maximal respiratory and spare respiratory capacity. The mHCs were isolated from ND, HFD-NS, and HFD-BMSCs mice at 45th week. All statistical data are represented as means ± s. *P < 0.05; **P < 0.01. Fig. S5. The flow cytometry analysis of mitochondrial ROS of different cells at 24 h and 48 h, respectively. HepG2-mito-GFP cells were cultured in FFA medium for 36 h and then co-cultured with BMSCs (FFA-BMSCs group) or not (FFA group) for another 24 h or 48 h. All statistical data are represented as means ± s. **P < 0.01. Fig. S6. Flow cytometry detects the double-stained cells. Labeled mHCs isolated form HFD mice were co-cultured with BMSCs-mito-GFP with different cell proportions (11:1/1:3/1:5/1:9/1:11) for 24 h, after which these cells were screened using 2.5 μg/mL puromycin. Q2 represents the double-stained cells. Table S1. Sequence information for the analysis of mt [file 13287_2021_2663_MOESM1_ESM.docx]

**Additional file 1**

**Bone marrow derived mesenchymal stem cell improves diabetes-associated fatty liver via mitochondria transformation in mice**

**Materials and methods**

**Isolation, culture and identification of BMSC**

The 4-6 week-old male C57BL/6 strain mice were euthanized utilizing CO2 followed by cervical dislocation and spray them with 70% ethanol. Previous study has well reported the marrow isolation procedure, and here we routinely followed the method to gather contents from femoral bone with small cuts via centrifuging at 10,000 🞨g for 15s at room temperature. Using a 25 G needle, pull the cell pellets up and down slowly to break up clumps and combine all the samples into a single 15 mL conical tube with 10mL of BMSC Culture Media (MarrowMAXTM, Gibco) per 500 μL of samples. Place a 70 μm filter on top of a 50 mL conical tube and pass the cell suspension through this filter in order to remove possible bone fragments. Marrows from 3 animals per experimental groups were pooled to yield a better representative ad reproducible cell population.

Calculate the cell number and plate thZem at 1.0 🞨10^6^ cells/ cm2 with 10 mL culture media into a 10 cm culture dish at 37℃ in an incubator with 5% CO_2_, trypan blue dyeing was recommended to evaluate the cell viability. And allow 72h of mixed culture for cells to attach followed by removing the media and replacing it with fresh media, from which the media was continually changed every two days and check for confluency. When the cells reach 80% confluency, they were ready for passaging.

The 6^th^ generation cells were subject to adipogenic and osteogenic induction for 14 days according to the protocols of MesenCult^TM^ Adipogenic Differentation Kit and Osteogenic Stimulatory Kit (STEMCELL), respectively. Then the cells were stained with Oil Red O and Alizarin Red S, respectively, for photographing. Flow cytometry performed the assay of surface markers of candidate cells. They were incubated with antibodies CD90.2, CD73, CD45, and CD34(BD Bioscience) for 20 min at 4 °C, washed on Lyse Wash Assistant (LWA, BD Bioscience) and acquired 20,000 events on a FACSAria IIIu (BD Bioscience). The data were analyzed using Flow JO software.

**Mouse primary hepatocytes (mHCs) isolation**

The mouse was anesthetized by intraperitoneal injection of anesthesia mix (3.75μL/g body weight. Final concentration for ketamine = 112.5mg/kg; for xylazine=22.5mg/kg). Place the mouse on the edge of the dissection tray, secure limbs using needles, and wet the fur thoroughly with 75% ethanol. Make a U-shaped incision through the skin and move the intestine to the right to reveal the portal vein and vena cava. Turn on the pump and let the warm perfusion buffer (487mL HBSS no Ca^2+^,no Mg^2+^, no phenol red; 0.5 mL 0.5 mM EDTA; 12.5mL 25mM HEPES) reach the needle. Here, the inferior vena cava was cannulated with the needle, then cut the portal vein with sterile scissors while immediately upon appearance of portal swelling. Next, plenty of blood would rush out of the portal vein and the liver will turn yellow-white within a few seconds. After completely washing out the blood, the liver is observed swelling an relaxation and ready for digestion with pre-warmed digestion buffer (50mL HEPES solution containing 1mL 25mg/mL Collagen II and 100uL 1M CaCl2) for 10-15min. Gently dissect the liver, remove the gall bladder, and then place the liver in the ice cold cell culture dish containing 10 mL DMEM. The live was ruptured sack with fine tip forceps n a few locations along the liver surface and gently release cells using a cell lifter. The released cells were filtered by a 200-mesh strainer and gathered them in a 50 mL tapered tube. The cells were pelleted and washed twice with DMEM medium by centrifugation at 50🞨g for 2 min at 4 ℃. Finally, prepared cells were subject to trypan blue staining to evaluate the cell viability, and then planted the living cells in 6-well plates at 4🞨10^5^ intensity per well.

**Quantitative reverse-transcription PCR (qPCR)**

Total tissue RNA was isolated using TRIzol, and cDNA was synthesized using the HiScript^®^ III all-in-one RT SuperMix (Vazyme, China). The qPCR was performed using the SYBR Green qPCR Mix (Aidlab Biotechnologies Co., Ld, China) in a Rotor-Gene-Q instrument (QIAGEN, Germany). The fold-change in gene expression was calculated using the 2^-ΔΔCt^ method, and β-actin was used as an internal reference. The primers used are listed in Additional file 1: Table S1 and S2.

**Western blotting**

Mouse tissues were homogenized and lysed in cold RIPA buffer in the presence of 1mM PMSF and protease inhibitor cocktail (Cell Signaling Technology) for 30min. The [supernatant](javascript:;) was collected after centrifugation at 12,000 rpm/min, and 4℃ for 20min. The concentration of total protein was determined using Bradford (Sigma, USA) mensuration. The mixture of the prepared protein and 5🞨 loading buffer was boiled for 15 min. All protein samples were homogenization in concentration and separated via sodium dodecyl sulfate-poluacrylamide gel electrophoresis (SDS-PAGE), transferred onto nitrocellulose membranes (Millipore, USA), and incubated with primary antibodies (Abcam, UK) against specific protein. Thereafter, the membranes were incubated with the appropriate horseradish peroxidase (HRP)-conjugated secondary antibodies (1 : 10,000, ZSGB-BIO, China), and the signals were detected using the SuperLumina ECL HRP Substrate Kit (Abbkine, USA). β-actin or GAPDH was used as an internal reference to assess protein levels. Three independent repetitive trials were performed.

**MDA assay**

Fresh liver was cut into small lumps to facilitate homogenization in RIPA (Beyotime, China) with 1 mM PMSF and protease inhibitor cocktail (Cell Signaling Technology, USA). The supernatant was collected from the lysate by centrifugation at 12,000 🞨 g for 10min at 4℃. The MDA Detection Kit (Beyotime) was used to detect MDA in the supernatant, and quantization was performed based on the the absorption peak at 532 nm.

**Hepatocyte MMP assay**

Adherent cells were washed twice with PBS and digested with 0.25% trypsin to prepare cell pellets via centrifugation at 800 🞨 g min for 4min. The cell pellet was resuspended in PBS, and MMP was performed according to the instruction of the JC-1 Kit (Invitrogen, USA). Finally, the fluorescence intensity of the suspension was assayed by flow cytometry at 491 nm excitation and 530 nm emission light.

**ROS detection**

Well-cultured cells were washed twice with PBS and digested with 0.25% trypsin. The cell pellet was prepared by centrifugation at 800 🞨 g for 4 min, and then resuspended in PBS containing 2 μL probe reagent 2',7' -dichlorofluorescein diacetate (Sigma, Missouri) or 10 μM MitoSOX™ Red Mitochondrial Superoxide Indicator (Invitrogen™, Massachusetts) to detect cell total ROS or mitochondrial ROS levels, respectively. After incubation in the dark at 37 ℃ for 30 min, 3mL PBS was added to the above mixture, followed by centrifugation at 800 🞨 g for 4min to discard the supernatant. Finally, the cell pellet was resuspended in 300 μL PBS and then subjected to flow cytometry for fluorescence intensity assay at 488 nm (total ROS) and 562 nm (mitochondrial ROS).

**Results**

**Establishment of T2D-associated NAFLD mouse model**

In this study, T2D model was established via 26-week HFD for further selection of T2D-associated ANFLD mice. According to the diagnostic criteria for type 2 diabetic mouse promulgated by WHO, it is determined as a T2D model characterized with over 40 g in consecutive 4 weeks, over 6.1mM of fasting blood glucose, and over 11.1 mM blood glucose after the intraperitoneal infection of glucose. In our study, the mean weight of HFD was significantly higher than ND group form 12^th^ week with *p*＜0.001(Additional file 1: Fig. S1 A). At 32^th^ week, we picked T2D mode mice based on above criteria plus over 45g in weight. GTT and ITT analysis both revealed that the blood glucose of HFD group was remarkably higher than ND group at the same time point (Additional file 1: Fig. S1 C). Also, fasting serum insulin of HFD notably exceeded that in ND group (*p*＜0.01), which indicated the T2D mice were suffering from hyperinsulinemia (Additional file 1: Fig. S1 B). ALT and AST were under investigation for the assessment of liver injury (Additional file 1: Fig. S1 D). As the conservative perspective, long term HFD (over 20 week) induce ensures the formation of NAFLD in mice combined with abnormally evaluated ALT and AST.

**Identification of BMSCs**

Here, we identified the BSMCs based on the BMSC identification criteria passed by the International Cell Therapy Association. BMSCs were isolated from the 4-6 week-old male C57BL/6 mice for continuous culture. To investigate the cell morphology, the 4^th^ passage of BMSCs was photographed and they exhibited a typical adherent growth with spindle shape. The adipogenic induction of 6^th^ passage BMSCs revealed the increase in cell volume, and lipid droplet aggregation proved by Oil Red O. Moreover, we could observe red stained area after in the 6^th^ passage BMSCs after osteogenic induction for 14 days (Additional file 1: Fig. S2 A). Flow cytometry described the 6^th^ passage BMSCs had positive surface markers (CD73, CD90.2) and negative surface markers (CD34, CD45) (Additional file 1: Fig. S2 B).

**
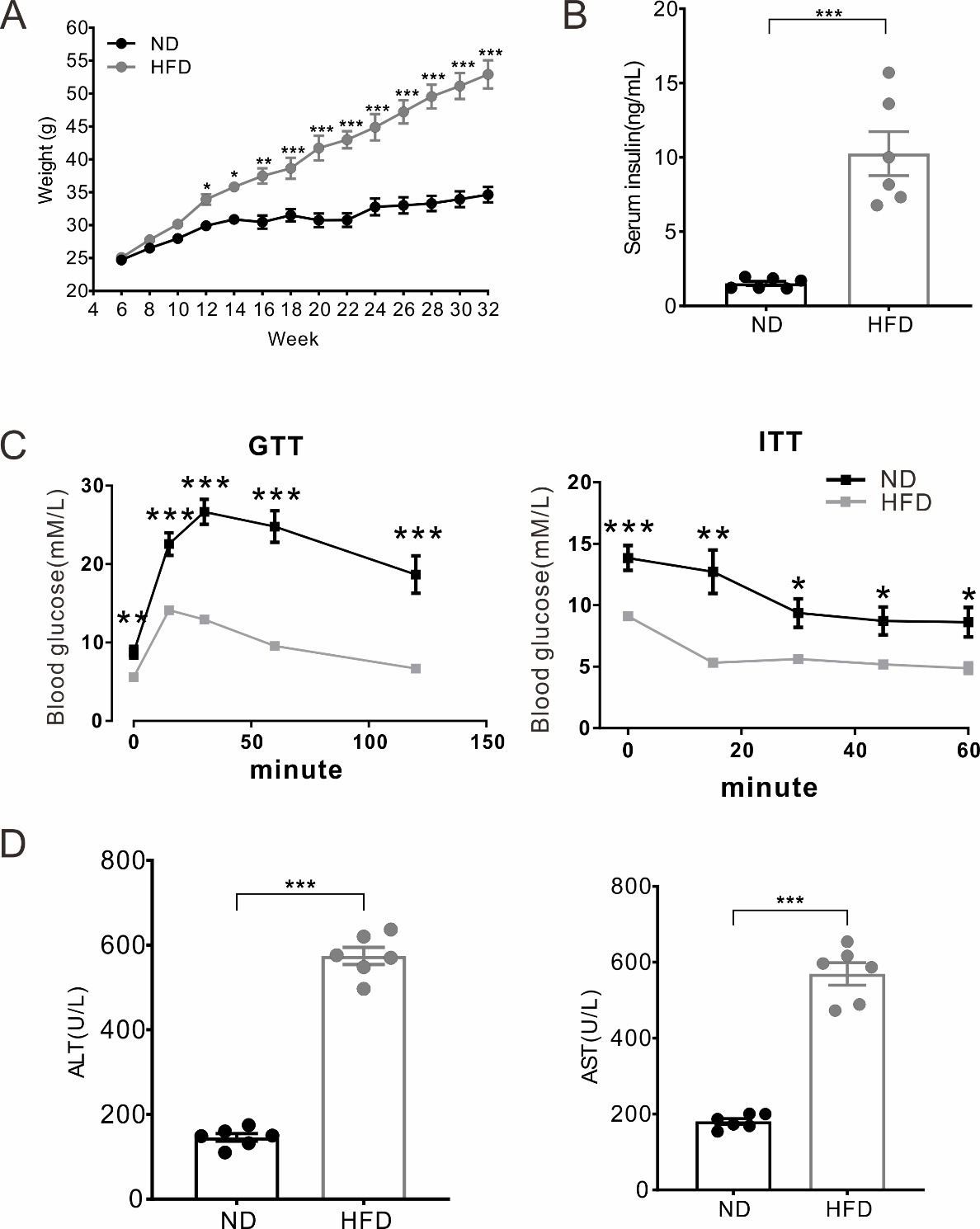
Additional file 1: Fig. S1. Establishment of T2D-associated NAFLD mouse model.** (A) The body weight monitoring of mice fed with HFD or ND from 6th week to 32nd week. (B) The detection of serum insulin at 32nd week. (C) The GTT and ITT assay at 32nd week. (D) The detection of serum ALT and AST at 32nd week. All statistical data are represented as means ± s. **P*＜0.05; ***P*＜0.01; ****P*＜0.001.

**Additional file 1:
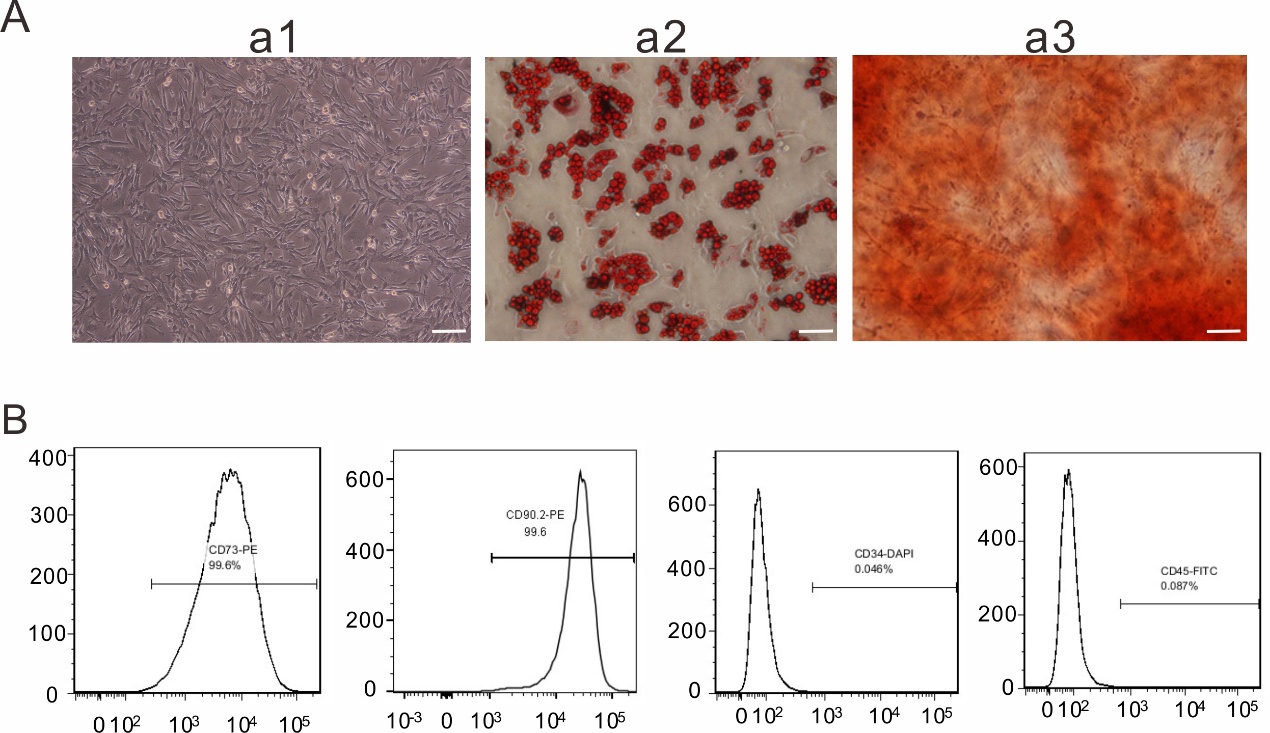
Fig. S2. The identification of BMSCs.** (A) The representative micrographs of the 4^th^ passage BMSCs (a1); Adipogenic differentiation of the 6^th^ passage BMSCs (Oli Red O stain of lipid droplets); Osteogenic differentiation of BMSCs (Alizarin Red stain for calcium). Scale bars 10 μm. (B) Representative flow cytometry analysis of cell-surface markers in the 6^th^ passage BMSCs. All BMSCs expressed cell markers included CD73 and CD90.2, however negative for CD34 and CD45.


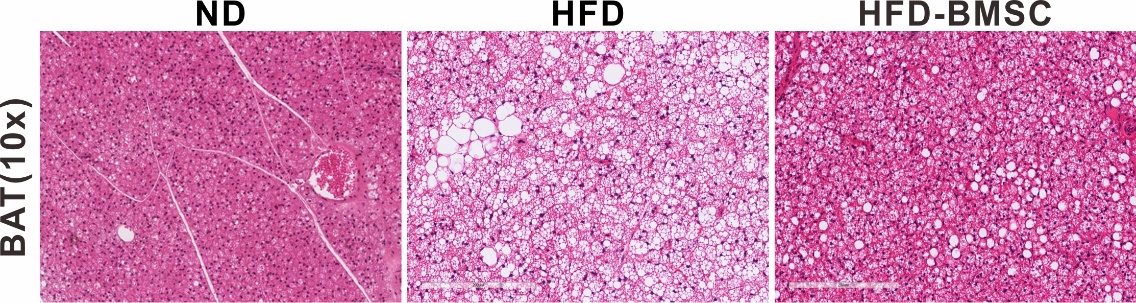


**Additional file 1: Fig. S3.** Representative images of HE stained BAT sections. ND, normal diet; HFD, high fatty diet; HFD-BMSC, high fatty diet mice with BMSC administration.

**Additional file 1:
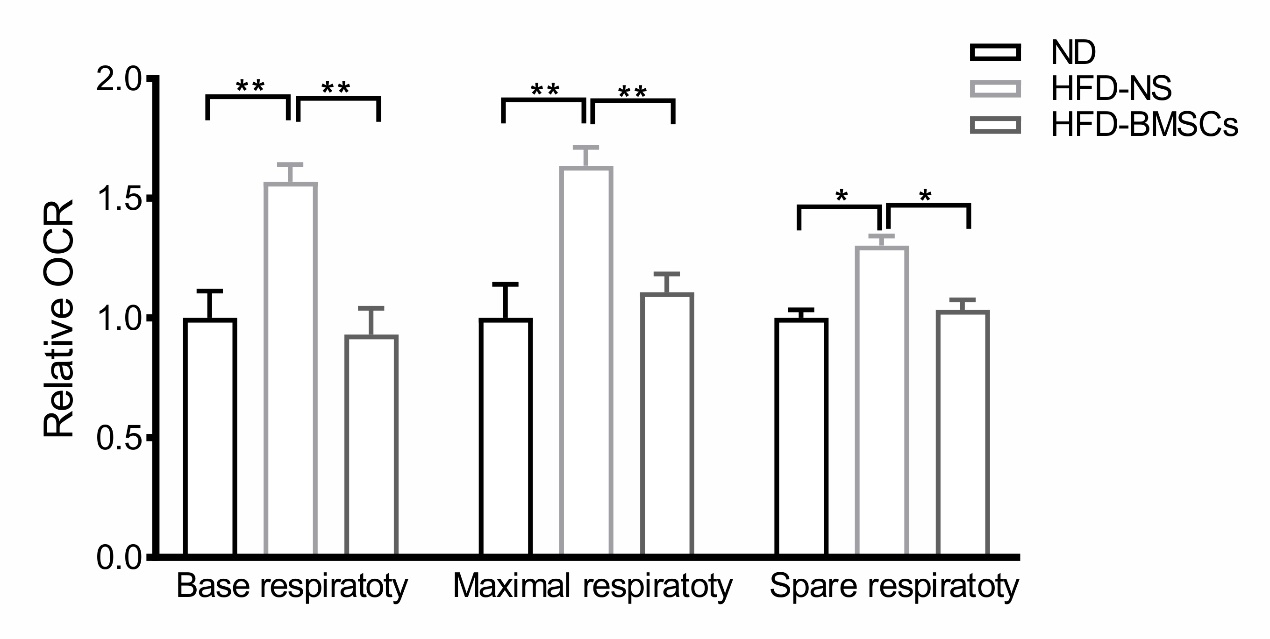
Fig. S4.** The calculations of mean OCR of the isolated mHCs at different stage corrected to basal OCR, maximal respiratory and spare respiratory capacity. The mHCs were isolated from ND, HFD-NS, and HFD-BMSCs mice at 45^th^ week. All statistical data are represented as means ± s. **P*＜0.05; ***P*＜0.01.

**Additional file 1:
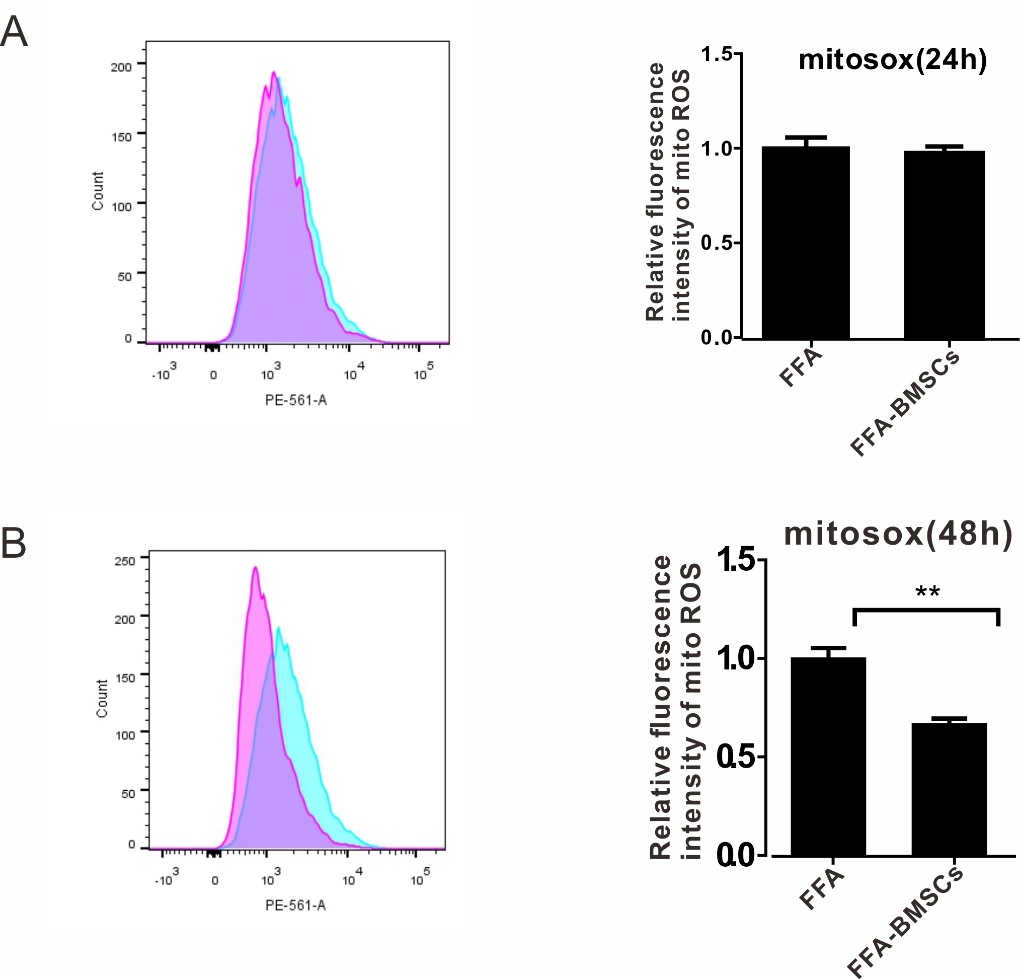
Fig. S5. The flow cytometry analysis of mitochondrial ROS of different cells at 24h and 48h, respectively.** HepG2-mito-GFP cells were cultured in FFA medium for 36 h and then co-cultured with BMSCs (FFA-BMSCs group) or not (FFA group) for another 24h or 48 h. All statistical data are represented as means ± s. ***P*＜0.01.

**Additional file 1:
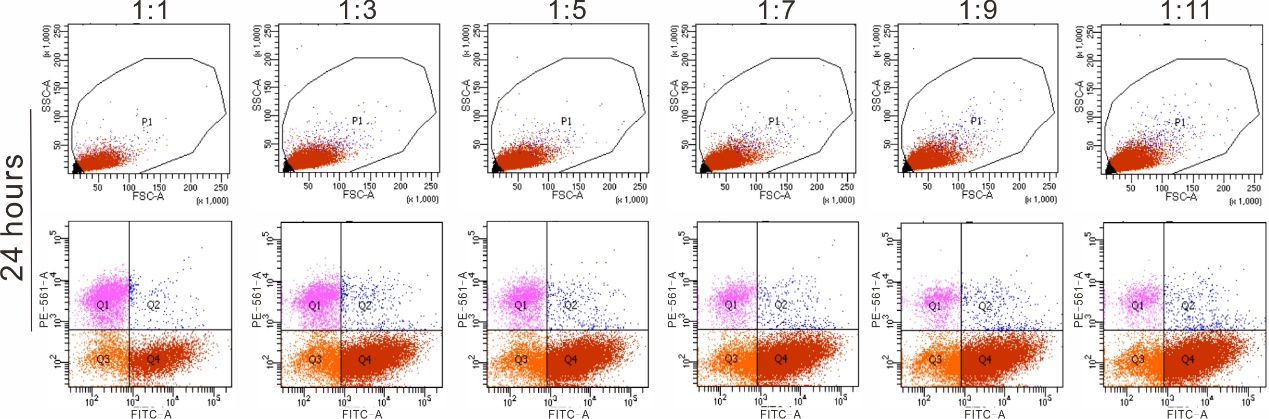
Fig. S6. Flow cytometry detects the double-stained cells.** Labeled mHCs isolated form HFD mice were co-cultured with BMSCs-mito-GFP with different cell proportions (11:1/1:3/1:5/1:9/1:11) for 24 h, after which these cells were screened using 2.5 μg/mL puromycin. Q2 represents the double-stained cells.

**Additional file 1: Table S1. Sequence information for the analysis of mtDNA/nDNA ratio.**

| Target genes | Forward primer (5’-3’) | Reverse primer (5’-3’) |
| --- | --- | --- |
| 16S rRNA | CCGCAAGGGAAAGATGAAAGAC | TCGTTTGGTTTCGGGGTTTC |
| ND1 | CTAGCAGAAACAAACCGGGC | CCGGCTGCGTATTCTACGTT |

**Additional file 1: Table S2. Sequence information of MQC-relevant genes.**

| Target genes | Forward primer (5’-3’) | Reverse primer (5’-3’) |
| --- | --- | --- |
| Fis1 | GCTGGTTCTGTGTCCAAGAGCA | GACATAGTCCCGCTGTTCCTCT |
| Drp1 | GCGAACCTTAGAATCTGTGGACC | CAGGCACAAATAAAGCAGGACGG |
| Mfn1 | CCAGGTACAGATGTCACCACAG | TTGGAGAGCCGCTCATTCACCT |
| OPA1 | TCTCAGCCTTGCTGTGTCAGAC | TTCCGTCTCTAGGTTAAAGCGCG |
| PGC-1α | GAATCAAGCCACTACAGACACCG | CATCCCTCTTGAGCCTTTCGTG |
| Tfam | GAGGCAAAGGATGATTCGGCTC | CGAATCCTATCATCTTTAGCAAGC |
| Nrf2 | CAGCATAGAGCAGGACATGGAG | GAACAGCGGTAGTATCAGCCAG |
| β-actin | CATTGCTGACAGGATGCAGAAGG | TGCTGGAAGGTGGACAGTGAGG |
